# A Perspective Discussion on Rising Pesticide Levels and Colon Cancer Burden in Brazil

**DOI:** 10.3389/fpubh.2017.00273

**Published:** 2017-10-16

**Authors:** Sergio Akira Uyemura, Helga Stopper, Francis L. Martin, Vinicius Kannen

**Affiliations:** ^1^Department of Toxicology, Bromatology, and Clinical Analysis, University of São Paulo, Ribeirao Preto, Brazil; ^2^Department of Toxicology, University of Wuerzburg, Wuerzburg, Germany; ^3^School of Pharmacy and Biomedical Sciences, University of Central Lancashire, Preston, United Kingdom

**Keywords:** agriculture, Brazil, colorectal cancer, International Agency for Research on Cancer, pesticides

## Abstract

Agriculture is a mainstay of many developing countries’ economy, such as Brazil. According to the Food and Agriculture Organization of the United Nations, Brazil is the major global consumer of pesticides. Irrespective of the fact that the International Agency for Research on Cancer suggests that pesticides promote human cancer risk, a prospective study reports that colorectal cancer (CRC) burden will increase in developing countries by approximately 60% in the coming decades. Here, we review the literature and public data from the Brazilian Federal Government to explore why pesticides levels and new cases of colon cancer (CC) are rising rapidly in the country. CC incidence is the second most common malignancy in men and women in the South and the Southeast of Brazil. However, while these regions have almost doubled their pesticide levels and CC mortality in 14 years, the amount of sold pesticides increased 5.2-fold with a corresponding 6.2-fold increase in CC mortality in Northern and Northeastern states. Interestingly, mortality from endocrine, nutritional, and metabolic diseases are rapidly increasing, in close resemblance with the pesticide detection levels in food. Taken together, we discuss the possibility that pesticides might alter the risk of CC.

## Introduction

The International Agency for Research on Cancer (IARC) reported recently that several pesticides increase the risk of cancer in humans (1, 2). The causal relationship between environmental factors and cancer has been intensively investigated by scientific researchers since Sir Percival Pott’s findings over 200 years ago. Pott correctly linked chronic exposure to soot, which contains high levels of carcinogenic polyaromatic hydrocarbons, with the high incidence of scrotal squamous cell carcinoma in British chimney sweeps. Because German chimney sweeps wore tight-fitting clothes, they had the lowest risk for that cancer (3). Another example is that cancer levels did not significantly impact on mortality rates in comparison with heart problems in the United States (US) in 1900, according to the US National Center for Health Statistics. A half century later, death rates remained three times higher for heart-associated diseases compared to cancer. In the next 63 years, relative heart disease mortality rates decreased to the point that they are on par with cancer (4) levels. Might environmental factors underlie such fluctuations in chronic disease levels in humans?

An interesting report on mesothelioma burden, which is an asbestos-related lung cancer, helps to clarify this question. Although exposure to asbestos reached its highest levels in the United Kingdom by 1953, the maximum mesothelioma mortality was predicted to occur only 63 years later (5). This long latency time makes it difficult to identify carcinogenic environmental threats. Moreover, today’s real-world environment presents an unpredictable complexity for human exposure to genome-damaging and/or epigenome-modifying compounds that might give rise to cancer (6). Alongside overnutrition and sedentary lifestyle, some reports suggest that 75% of cancers are related to chronic exposure to endogenous and/or exogenous environmentally generated factors (6, 7).

It is worth noting that pesticides can contaminate red meat, as well as other food sources (i.e., fruits, vegetables, grains, fish, poultry), adding a further potential carcinogenic source to the possible mutagen content that a human meal might have (8). Lodovici et al. evaluated the genotoxic potential of 15 pesticides found in Italian foods. Only diphenylamine and chlorothalonil generated DNA damage in hepatocytes (9). In carcinogen-exposed rats, food containing captan and propineb increased cancer risk in the thyroid, kidney, urinary tract, and bladder (10). Lee et al. studied 49,980 pesticide applicators and reported that alachlor probably increased the burden of lymphohematopoietic cancers in this cohort (11). However, chemical interactions that induce cancer have been traditionally overlooked when only the carcinogenic potential of individual compounds is explored.

Analyzing 3,800 serum samples from 35 countries, Wang et al. suggested that pesticides increased the mortality rates associated with hepatocellular carcinoma, lung adenocarcinoma, and colon cancer (CC) in different human populations (12). In Spain, Luzardo and colleagues investigated whether the concentrations of pesticides in different kinds of meat could impact on cancer risk. These authors suggested that meat containing high-pesticides levels might increase the risk of cancer (13). Greenson and colleagues investigated 860 Egyptians who were either healthy or were diagnosed with colorectal cancer (CRC). It was shown that either eating pesticide-containing food or exposure to industrial pollution increased the CRC risk in that human population (14). In rats, Hong et al. observed that pesticides increased the CC risk (15).

The Hallmarks of Cancer model demonstrates that DNA damage is not the only event leading to malignancies and that carcinogen-induced changes in several other protective elements are also needed (16). Thus, new considerations about the carcinogenic or cancer-promoting effects of environmental chemicals must be taken into account (17). It seems possible that xenobiotics do not only induce somatic genomic mutations and epigenetic changes, but they also may disrupt the neuroendocrine system (18–22). Based on 34,205 cancer cases and 1,832.969 control subjects, Alarcón and colleagues suggested that high exposure to pesticides might increase the risk of all cancers, excluding Hodgkin and non-Hodgkin lymphomas (23). Interestingly, organochlorines and organophosphates induce non-genotoxic effects in different murine models (24, 25) although some organochlorines, namely chlorpyrifos, methyl parathion, or malathion, appear to induce oxidative stress (24). In Egypt, Soliman et al. observed that rather than control subjects, CRC patients exhibited higher serum organochlorine pesticide levels (26). Indeed, in 2013, Meyer and colleagues revealed that pesticides could be related to increased non-Hodgkin’s lymphoma mortality found in Brazil (27). Notably, it has been predicted that a 60% increase in the global burden of CRC will occur in developing countries by 2030 (28). Herein, we review how pesticides may alter the risk of CC.

## The Relationship Between Pesticides and the CC Risk: A Developing Country as an Example

Hannun and colleagues showed that environmental factors directly impact on cancer risk (29). Nielsen and colleagues explored the genome and transcriptome in 1,082 tumors revealing that the metabolism of arachidonic acid and xenobiotics determines cancer patient survival (30). One should not forget that DNA damage is not the only mechanism by which xenobiotics generate cancer (31, 32). Exposure to xenobiotics have been reported to induce oxidative stress, genomic damage, and a high expression of some cancer-related genes in subjects carrying a higher number of risk alleles to cancer (33). Considering the etiology of CC, ingestion is probably the main route by which carcinogenic chemicals accessing the human body cause this malignancy (17). Interestingly, Avancini et al. detected pesticides in bovine milk in the Brazilian Midwest region (34). High-pesticide levels have also been found in human milk in several Brazilian regions since 1992 (35–37).

In Western countries, CRC is the third commonest cancer and the second leading cause of cancer-related death (38). The incidence of driver mutations in CRC is typical of solid cancers that are driven by an ever-increasing age-related mutational burden, as approximately 90% of patients are 50 years or more at the time of diagnosis (39, 40). Current disease statistics indicate that CRC incidence is exhibiting a demographic shift to patients who are <50 years old (40), which may be an indication that the contribution of environmental factors is increasing. It seems that the intake of specific foods modulates the CRC risk. For instance, drinking less alcohol (0.5 g/day instead of 70 g/day) reduced CRC risk ~56-fold, whereas increasing meat intake ~2.6-times might promote CRC 1.4-fold (41). Indeed, the outlook for the incidence of this malignancy is bleak; as the world’s population increases and countries modernize, reports predict up to a 60% increase in the worldwide burden of CRC in developing countries such as Brazil by 2030 (28).

The Brazilian National Cancer Institute predicted that ~34,280 new CRC cases would be diagnosed in the country by 2016. Thus, CRC was expected to be the third and the second most common malignancy in men and women, respectively. Indeed, CRC is the second most often diagnosed malignancy for both sexes in the Brazilian Southeast. In 2016, the Southern Brazilian region surpassed the Southeast states in CRC cases per 100,000 inhabitants. While cancer mortality almost tripled in 30 years, CC-related deaths were ~4-time less frequent in 1984. Although the South and the Southeast regions endured a ~4-fold increase in these mortality rates, deaths from CC increased ~5.2-fold in the Northern and the Northeastern states. We may better understand these facts knowing that the Brazilian population only grew ~1.7-fold in the same time-period.

Brazil has become the leading worldwide user of pesticides from 2008 onward. For instance, Brazilians imported 1,132-fold more pesticides in 2014 than in 1984. Within the country, the amount of pesticides sold increased from 162,462 to 508,557 t in the last 16 years. It must also be noted that pesticides were sold at an amount of ~19 kg/km^2^ in 2000, but that more than tripled within the next 14 years (~59.5 kg/km^2^). While the Southern region more than doubled the amount of pesticides bought per year [from ~89 kg/km^2^ (2000) to ~221 kg/km^2^ (2014)], this increase was ~7-times higher in the Northern states [from ~0.64 kg/km^2^ (2000) to ~4.5 kg/km^2^ (2014)]. This led us to inquire whether pesticides also contaminate food in Brazil.

The Brazilian National Health Surveillance Agency (Anvisa) annually reports the detection of pesticides in food. From 2001 to 2007, this agency revealed that ~13% of food sources did not comply with the safety standards for human use. In 2009, 3,130 food samples were analyzed, revealing that 29% of them contained pesticides above safety levels. This means that 744 samples contained illegal pesticides (IP), 88 samples had pesticide concentration above the maximum residue level (MRL), while 75 other samples had either IP or pesticide levels higher than MRL. A similar scenario was repeated in 2010, in which banned pesticides were detected in 605 out of 694 contaminated food samples. Astonishingly, between 2011 and 2012, the same agency found that 36% of analyzed food samples were unsafe for humans. Specifically, 32% of unsafe food samples contained IP, 2.3% of them did not comply with MRL, and 1.9% had both irregular characteristics. The latest report (2013–2015) showed that 20% of analyzed food samples contained either prohibited pesticides or contamination levels above safety standards. While soy plantations, only in 2011, have used ~341.2 million liters of all 852.8 million liters of agrochemicals sprayed on Brazilian crops, Anvisa did not show any analysis for soy contamination by pesticides in its reports.

Douglas and Tooker have recently reported increased usage of pesticides in plantations of soybeans and maize throughout the last decade in the US (42). Another research group suggested that most farmhouses are contaminated with pesticides (43). It has also been shown that soy sauces and related products contain significant levels of a pesticide (44). Although another report did not support the notion that soy-related manufactured products contained pesticides, such chemicals were largely found in soy protein isolated from genetically modified soybeans (45). In Argentina, pesticides contamination seems to have reached the groundwater (46).

**Figure 1:**
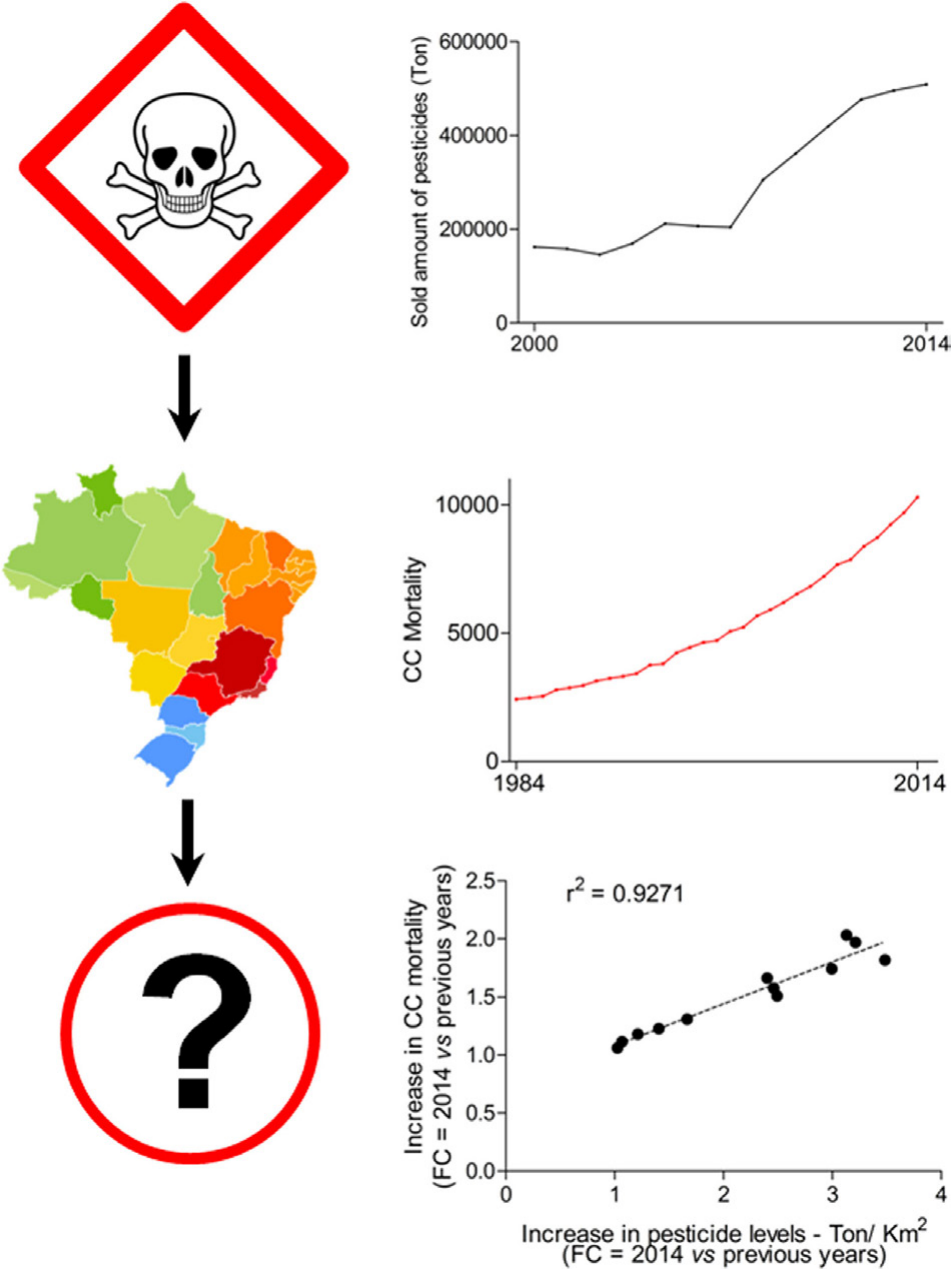
Correlation between colon cancer (CC) mortality and pesticides levels in Brazil. Quantity of sold pesticides per area of Brazilian regions (km^2^). Data from the Brazilian Institute of Environment and Renewable Natural Resources (IBAMA) is publically available (link: http://dados.contraosagrotoxicos.org/pt_PT/dataset/comercializacao-ibama-2014). Area of each Brazilian was consulted at the Brazilian Institute of Geography and Statistics website (IBGE; http://www.ibge.gov.br/). Cancer and CRC mortality in Brazil (1984–2014). Mortality numbers for cancer and CRC in Brazil. Dataset was downloaded from the website of the Ministry of Health (link: http://www2.datasus.gov.br/DATASUS/index.php?area=0205). The increase in CC mortality and pesticides levels [fold change (FC) was determined comparing data from 2014 against the 13 previous years] throughout 14 years were analyzed by the Pearson’s *r* test. The *r* squared (*r*^2^) value is shown.

The National Toxic-Pharmacological Information System (SINITOX) collects and analyzes all cases of acute intoxication and poisoning in the country each year. These reports showed that 78,623 Brazilians endured acute pesticide-related poisoning, from which 2,524 people died from 1999 to 2013. The highest numbers were observed between 2005 and 2007. Although Brazil may lack a follow-up on how chronic exposure affects its population, mortality by endocrine, nutritional, and metabolic diseases (ICD-10; E00-E90) more than doubled throughout the same period (1996–2014). Having all these data together directed us to determine whether the increase in CC mortality might be correlated with pesticides levels in Brazil (Figure 1). Collectively, our data seem to suggest that pesticides could critically influence the risk of CC in Brazilians.

## Potential Mechanisms of How Pesticides Alter the CC and CRC Risks

Currently, the carcinogenic potential of pesticides is a controversial issue. For instance, in Turkey, farmers underwent a CRC screening that revealed a reduced risk to develop this type of cancer (47). However, in Egypt, high serum pesticide levels were detected in patients diagnosed with CRC (26). Another Egyptian report suggests that food containing pesticides might increase CRC risk (14). Notably, following the Vietnam War, Korean soldiers exposed to the pesticide Agent Orange exhibited high rates of CRC (48, 49). Thus, we must consider another study showing that pesticide-exposed fish either had no impact on CRC risk, or decreased the risk for CRC (50). In this context, it is interesting that experiments with rats exposed to pesticides but treated with fish oil revealed that the pesticides increased the CC risk and reduced the chemoprotective effects of that oil (15). Arrebola et al. reported that food containing pesticides increased the risk of breast cancer in Tunisian women (20). Exposure to pesticides in an American farmer population enhanced the risk of obesity-related CRC (19).

Some molecular studies seem to support the idea that pesticides promote the risk of cancer. A pesticide and xenobiotic named endosulfan promoted colon inflammation with concomitant upregulation of β-catenin and interleukin-6 expression (51). Another interesting report showed that the pesticide chlorpyrifos activates the EGFR/ERK1/2 growth signaling pathway to promote CRC development (52). These facts may suggest the hypothesis that at least some pesticides act as tumor promoters in the rapidly dividing colonic epithelial cell population (53). Instead of DNA-damaging effects induced by cancer initiators, endogenous and/or exogenous cancer promoters are classically determined to lead mutated cells toward clonal expansion, enabling them to collect further genomic changes by either high proliferative activity or new carcinogenic hits (54). Rather than binding to DNA, a cancer promoter usually activates transcriptional and epigenetic mechanisms that induce proliferation but inhibit apoptosis (54, 55). Such mechanistic activity has long been known to induce proliferation intrinsic errors leading to mutations and the development of CRC (53, 56, 57).

Environmental pollutants, such as pesticides, might impair neuroendocrine functions promoting chronic inflammation that facilitates cancer initiation and progression (58–60). It should be considered that the gut is the largest endocrine organ in the human body (61). With ~500 million neurons (62), intestines not only synthesize more than a 100 hormones by endocrine cells but also harbor ~70% of immune system activity in the body (61, 63). This impacts on many bodily functions in either physiological or pathological condition, including cancer development (61, 64). Another interesting fact is that immune cells seem to interact with environmental inputs through the intestinal microbiome balancing the host immunity (65). Impairments in this fine-tuned mechanism by diet are one of many examples that might enhance the risk of CC and CRC (66). There seems to be some evidence that xenobiotics alter the intestinal microbiome increasing the risk of CC and CRC (67–69).

Human exposure to contaminated arsenic soil seemed to enhance cancer mortality (70). In mice, adding arsenic to their drinking water altered the gut microbiota and metabolism (71). Dheer et al. have reported that chronic exposure to arsenic changed the composition of intestinal microbiota, increased bacterial spores, altered the intestinal and hepatic nitrogen metabolism, and enhanced pathogenic arginine metabolites in blood circulation (72). Gut dysbiosis can promote CRC through either an immune system deregulation, such as that related to chronic inflammation and oxidative stress or direct-DNA damage and genomic instability (68, 69). Gram-negative bacteria seem to induce direct-DNA damage and genomic instability releasing the cytolethal distending toxin and colibactin, which damage the DNA and trigger the phosphorylation of histone H2AX. This phosphorylated histone activates a transient G2/M cell cycle arrest and cellular swelling through the ataxia-telangiectasia mutated-checkpoint kinase 2 signaling pathway (68). On the other hand, an imbalance between intestinal Gram-positive and -negative bacteria can promote a microorganism-driven tumor-initiating inflammatory condition in NF-κB- and IL10-dependent mechanisms (68, 69). Thus, a microbial translocation across the intestinal epithelial barrier seems to foster the malignant outgrowth, since it enhances even further recurrent inflammation-related cancer (69).

Given that tumor cells are initially immunogenic, dendritic cells survey cancer-initiating cellular hot spots for antigen expression after somatic mutations are established, which are then processed into major histocompatibility complex (MHC) classes I and II within regional lymph nodes. MHC II is presented to CD4+ T lymphocytes, whereas MHC I activates CD8+ T cells. The balance between MHC I and II pathways is thus crucial for determining the lytic cycle against malignant cells because it modulates the differentiation of T cells into cytotoxic T lymphocytes. Subsequently, immune cells must overcome immune checkpoints and cancer immunosuppressive networks to maintain their cytotoxic potential against tumor cells (73). While tumor cells are initially immunogenic, which means immunosurveillance is highly activated and able to block malignant development, tumor changes promoted by immune-editing drive cancer cells to silence the immune system through chronic antigenic stress. Indeed, chronic inflammation can facilitate early malignant steps promoting the immunosurveillance to target cancer-initiating senescent cells, which provides a substantial growth advantage by cellular selection in the incipient tumor (21). Thompson et al. have hypothesized that xenobiotics could disrupt the stromal–epithelial interactions targeting the immune system, from which cancer risk could be increased in exposed subjects (67). Lyerly and colleagues have recently suggested that exposure to xenobiotics might impair the complex activity of immunosurveillance against cancer, a fact that could enhance tumor incidence in the general population (73). Kleinstreuer and colleagues believed that xenobiotics would provide a growth advantage to cancer cells by inducing angiogenic changes in the tissue microenvironment (58), which could happen together with cancer-related immune dysfunctions (58, 67). Hence, human exposure to pesticides possibly results in deep tissue changes beyond the well-characterized carcinogenic events in epithelial cells. However, current scientific methods have a few limitations that should be considered.

## Scientific Limitations to Study the Carcinogenic Effects of Pesticides

First of all, environmental levels of several human-made pollutants might lack an immediate carcinogenic effect, such as DNA damage (5). In the multi-staged CC etiology, xenobiotics are one of the unaccountable confounding agents that preclude direct causative linkage between exposure to pesticides and CC risk. In further evidence, Barupal and colleagues have recently shown how little evidence is available to determine causative linkage between xenobiotics and cancer. Analyzing about 6,000 chemicals with chemoinformatic tools, they identified that the effects of only 8 out of 980 pesticides had been explored in cancer (74). It is known that there is a large number of xenobiotics, some with a bioaccumulative potential, that they affect the metabolism of exposed individuals differently, have a molecular mass <1,000 Da, share insignificant chemical similarities, can act as either agonists or antagonists of steroid hormone receptors, their source determines which tissue they might target, their exposure levels and chemical combinations might vary even within the same geographic area, and their combinations and levels might induce a plethora of different effects (33, 54, 75–78).

In this regard, the lowest-observed-adverse-effect level test has been widely applied in animal models to reveal, together with either linear extrapolation or benchmark dose modeling, side effects of untested chemicals that will be used by humans. Given that such methods cannot adequately forecast whether the synergism between and amongst low-dose mixtures of single compounds promotes cancer, several studies have applied non-linear dose-response calculations. It has been revealed that the interaction of low-dose chemical exposures promoting cancer does not necessarily have to occur simultaneously or continuously, but they can indeed act sequentially or discontinuously in a far more potent carcinogenic fashion than any single chemical exposure could be (79).

The Adverse Outcome Pathway (AOP) concept has also provided novel insights that connect the pathological basis of a disease to risk assessment. AOP applies high-throughput screening assays to associate the effects of different chemical exposures with major targets and pathways within the Hallmarks of Cancer framework, which provides the first linkage among a chemical exposure, a direct molecular initiating event, and an adverse biological malignant outcome. Although it has been extremely difficult to test potential effects of low-dose mixtures of single compounds for cancer risk, response addition and dose addition are mathematical strategies that help in the assessment. Whereas response addition assumes that different chemicals have the same outcome through various modes of action, dose addition can be applied when distinct compounds have a similar activity (79).

## Conclusion and Future Perspectives

Together with several lines of scientific evidence that the cancer risk may be altered by decades of exposure to environmental factors (5, 6, 76), the IARC has emphasized the carcinogenic effects of pesticides for a few years now (1, 2, 80–82). There also seems to be a potential indication that xenobiotics alter genomic repair and inflammatory mechanisms to impact on cancer (16, 17, 21, 55, 58–60, 67, 73, 83–86). Mixture effects (87) and low-dose effects (88) remain to be elucidated. These facts might lead to the point that acute pesticide poisoning is not the main route by which they impact on the risk of cancer. As much as pesticide usage has enabled the increase of global food production and prosperity, now may have come the time to re-evaluate strategies in farming, to mitigate the increasing risk of CC and other cancer types.

## Collection of Public Data

CRC burden^1^ and CC mortality (from 1984 to 1995, http://tabnet.datasus.gov.br/cgi/deftohtm.exe?sim/cnv/obt09uf.def [select: Year, Capitulo CID-9 (IX), Categoria CID-9 (153)]; from 1996 to 2014, http://tabnet.datasus.gov.br/cgi/deftohtm.exe?sim/cnv/obt10uf.def [select: Year, Capitulo CID-10 (XI), Categoria CID-10 (C18)])^2^, as well as mortality by endocrine, nutritional, and metabolic diseases, were collected from the database of the Ministry of Health. The same source provided data for the incidence of other types of malignancies. Data on pesticides were also collected from the UN^3^. The quantity of sold pesticides within the country was downloaded from the website of the Brazilian Institute of Environment and Renewable Natural Resources^4^^,^^5^. Data on pesticides poisoning and mortality were obtained from the National Toxic-Pharmacological Information System^6^. The Brazilian National Health Surveillance Agency website was consulted for data on food contamination by pesticides^7^. Complementary data on pesticides and area (Km^2^) were collected from the Brazilian Institute of Geography and Statistics (see text footnote 5). Basic calculations [fold change (FC), percentage, and normalization (weight/area)] were performed. Figure 1 shows data from the Pearson’s *r* test analysis. The increase in CC mortality and pesticide levels was calculated in FC (values from 2014 against previous years they were analyzed within each different Brazilian regions, as well as for the whole country). Then, we statistically analyzed the correlation between values by the Pearson’s *r* test in the GraphPad Prism 5 software (Graph Pad Software Inc., US). A strong correlation showed *r* squared (*r*^2^) close to 1, and *P* < 0.05.

## Author Contributions

Study concept and design, acquisition of data, statistical analysis, drafting the first version of the manuscript, obtained funding, and study supervision: VK; analysis and interpretation of data and critical revision of the manuscript: VK, HS, FM, and SU.

## Conflict of Interest Statement

The authors declare that the research was conducted in the absence of any commercial or financial relationships that could be construed as a potential conflict of interest.

## Footnotes

^1^http://www.inca.gov.br/estimativa/2016/estimativa-2016-v11.pdf.

^2^http://www2.datasus.gov.br/DATASUS/index.php?area=0205.

^3^http://www.fao.org/faostat/en/#data/RT.

^4^http://dados.contraosagrotoxicos.org/pt_PT/dataset/comercializacao-ibama-2014.

^5^http://www.ibge.gov.br/.

^6^http://sinitox.icict.fiocruz.br/dados-nacionais.

^7^http://portal.anvisa.gov.br/en/programa-de-analise-de-registro-de-agrotoxicos-para.
